# Leptospirosis on the edge: a case report of critical multi-organ involvement

**DOI:** 10.1186/s12879-026-12856-z

**Published:** 2026-03-25

**Authors:** Hiba Merhi, Chirine Mollaei, Bilal Damen, Zeinab Mawla, Ali Raad

**Affiliations:** 1 Department of Pulmonary & Critical Care, Faculty of Medical Sciences, Lebanese University, Beirut, Lebanon; 2 Department of Pulmonary & Critical Care, al Rassoul Al Aazam Hospital, Beirut, Lebanon

**Keywords:** Severe leptospirosis, Weil’s disease, Jaundice, Multi-organ dysfunction, Case report

## Abstract

Leptospirosis is a bacterial zoonotic disease caused by *Leptospira* species, commonly transmitted through direct or indirect contact with contaminated water, soil, or animal urine. Although typically a self-limiting illness, severe forms of leptospirosis (Weil’s disease) can lead to multi-organ dysfunction, including hepatic and renal failure, hemorrhagic manifestations, and altered mental status. This case report describes a 52-year-old male who presented with severe jaundice, nausea, vomiting, diarrhea, and multi-organ dysfunction, including acute kidney injury, liver failure, and thrombocytopenia, following a recent history of fever and self-medication. Initial investigations revealed elevated inflammatory markers, liver enzymes, renal dysfunction, and thrombocytopenia, raising suspicion for leptospirosis. Empiric treatment with doxycycline was initiated, and the diagnosis was confirmed via positive *Leptospira* IgM (Enzyme-Linked Immunosorbent Assay - ELISA) testing. Despite the severe nature of the illness, the patient showed gradual recovery following supportive care, including hemodialysis and liver protection strategies. This case underscores the importance of early recognition and empirical treatment of leptospirosis in severe cases, as timely intervention can significantly improve outcomes and highlights the need for public health strategies targeting reservoir control and community awareness to avoid its outbreak in the community. Early diagnosis and intensive supportive care, including organ-specific management, are critical for mitigating the high morbidity and mortality associated with multi-organ dysfunction in leptospirosis.

## Introduction

Leptospirosis is a bacterial zoonotic disease caused by *Leptospira* species, which primarily affects humans following direct or indirect contact with contaminated water, soil, or animal urine. The disease is endemic in tropical and subtropical regions, where it is prevalent due to environmental factors like poor sanitation and flooding. The *Leptospira* bacteria are transmitted through broken skin or mucous membranes when exposed to water or soil contaminated by the urine of infected animals [1].

Globally, leptospirosis is endemic in tropical and subtropical regions, particularly in areas with inadequate sanitation, increased rainfall, and high human-animal interaction. The World Health Organization (WHO) estimates that more than 1 million cases of leptospirosis occur annually, with approximately 59,000 deaths worldwide [2]. In Lebanon, while leptospirosis cases are relatively rare, the risk increases during periods of flooding or heavy rains that may contaminate water sources with the bacteria. Multi-organ dysfunction occurs in approximately 5–15% of severe cases of leptospirosis, with renal failure being the most common complication [3]. This case highlights the presentation of severe leptospirosis with multi-organ dysfunction in a 52-year-old male patient, emphasizing the diagnostic challenges, immunopathogenesis, treatment, and recovery process.

## Case presentation

A 52-year-old male presented with jaundice, nausea, vomiting, and multiple episodes of diarrhea. His symptoms began one week prior to admission with rhinorrhea, fever, and cough, for which he self-medicated with over-the-counter medications, including acetaminophen and herbal remedies.

Epidemiological Context: The patient was previously healthy, nonsmoker, nonalcoholic, had no history of prior surgeries, allergies, recent travel, or insect bites. No family history for any genetic diseases was reported. However, upon further questioning, the patient reported walking through stagnant water near his home following recent heavy rainfall two weeks prior to symptom onset, providing a crucial epidemiological link.

Clinical Findings: Upon arrival at the emergency department, the patient was normotensive, heart rate was around 60 beats per minute (bpm), SpO2 97% on room air, and febrile (39 °C). He was conscious, cooperative, and oriented. Physical examination revealed jaundice with icteric sclera, conjunctival suffusion, no significant murmurs, and clear lung fields. Abdominal examination was unremarkable, with mild tenderness, no guarding, or hepatosplenomegaly. Neurological examination was normal.

Initial Investigations: Initial laboratory investigations revealed leukocytosis (WBC 11,680/mm³ with 88.5% neutrophils), severe thrombocytopenia (platelets 23,000/mm³), and anemia (Hb 7.1 g/dL). Renal function tests showed acute kidney injury (creatinine 6.4 mg/dL, BUN 128 mg/dL). Liver function tests were significantly abnormal, with total bilirubin of 26.3 mg/dL (direct bilirubin 11.4 mg/dL), mild transaminitis (Aspartate Aminotransferase [AST] 74 IU/L, Alanine Aminotransferase [ALT] 39 IU/L), and hypoalbuminemia (2.5 g/dL). Inflammatory markers were elevated, including C-Reactive Protein (CRP 19.8 mg/L). Serum amylase was also markedly elevated (617 U/L). Hepatitis A IgM was negative.

Hospital Course: Given the worsening renal function and systemic involvement, the patient was admitted to the intensive care unit (ICU). Empirical broad-spectrum antibiotic therapy with piperacillin-tazobactam (2.25 g IV every six hours) was initiated after obtaining blood and urine cultures, which later returned negative.

On the second day of ICU admission, renal and hepatic function continued to deteriorate. Laboratory results showed worsening kidney injury (BUN 128 mg/dL, creatinine 7.33 mg/dL), increasing bilirubin levels (total bilirubin 31 mg/dL, direct bilirubin 26 mg/dL), and worsening inflammation (CRP 27 mg/L). Coagulation studies revealed a prolonged Prothrombin Time (PT 56 s), Partial Thromboplastin Time (PTT 37.6 s), International Normalized Ratio (INR 1.52), and elevated fibrinogen (876 mg/dL). Arterial blood gases indicated metabolic acidosis (pH 7.33, HCO3 14 mmol/L, PCO2 21 mmHg).

Clinically, the patient became confused, agitated, and oliguric, raising concerns for uremic encephalopathy and worsening metabolic acidosis. Hemodialysis was initiated following the insertion of a dialysis catheter, and vitamin K (10 mg IV) was administered to address coagulopathy.

Diagnostic Workup: Given the constellation of jaundice, acute kidney injury, thrombocytopenia, and altered mental status, an extensive workup was initiated to investigate infectious, autoimmune, hematologic, and toxicological etiologies. Infectious and autoimmune serologies included leptospirosis IgM, anti-mitochondrial antibody (AMA), anti-smooth muscle antibody (ASMA), antinuclear antibody (ANA), hepatitis A and E serologies, Weil-Felix, and Widal/Wright tests. Hematologic and metabolic investigations included a peripheral smear, vitamin B12, folic acid, reticulocyte count, iron studies, HFE gene mutation, ammonia level, and serum electrophoresis. A toxicology screen was also conducted. Imaging studies included an abdominal Doppler ultrasound, which showed normal kidney size and echotexture, no biliary duct dilatation, and no ascites. Liver elastography revealed normal stiffness (4.24 kPa, F0), and the hepatic parenchyma appeared mildly heterogeneous, suggestive of an infectious or inflammatory process.

Management: Given the clinical suspicion of leptospirosis (Modified Faine’s score of 32), empiric doxycycline (100 mg IV every 12 h) was initiated while awaiting confirmatory testing. Additional supportive therapies included N-acetylcysteine (NAC) and rifaximin for possible hepatic encephalopathy, ursodeoxycholic acid for cholestasis, and enteral nutrition via an orogastric tube due to worsening neurological status.

Despite initial stabilization, the patient developed progressive drowsiness, confusion, and persistent vomiting over the next 48 h. A peripheral smear revealed thrombocytopenia, leukocytosis, neutrophilia, immature myeloid cells, target cells, and rouleaux formation. Serum ammonia was elevated (123 µmol/L), and iron studies showed high ferritin level. Molecular testing for JAK2, CALR, MPL, and BCR-ABL mutations was negative, ruling out myeloproliferative disorders. PCR for viral and bacterial meningitis was also negative. Due to persistent melena and worsening anemia despite transfusions, an upper gastrointestinal endoscopy was performed to investigate potential gastrointestinal bleeding.

## Definitive diagnosis and recovery

On day 10 of hospitalization, the *Leptospira* IgM test (ELISA) returned positive, confirming leptospirosis as the underlying cause of multi-organ dysfunction. (Note: Molecular testing for *Leptospira* PCR was unavailable at our center, and the serology sample was processed externally). Over the next few days, the patient’s neurological status improved, with a gradual return to full consciousness (GCS 15/15 by day 12). The orogastric tube was removed, and he was transitioned to an oral diet.

Laboratory parameters also showed improvement. Creatinine levels fluctuated but ultimately improved from 8.78 mg/dL (day 2) to 3.89 mg/dL (day 12). Bilirubin levels decreased from 31.1 mg/dL on admission to 7.5 mg/dL by day 12, with a corresponding decline in direct bilirubin from 26.2 mg/dL to 6.6 mg/dL (Table 1). Transaminase levels remained below 100 IU/L throughout hospitalization. Following continued clinical and biochemical improvement, the patient was discharged home in stable condition with plans for outpatient follow-up and supportive care.

**Table 1:** Evolution of key laboratory parameters

Parameter	Admission (Day 1)	Peak Severity (Day 2)	Discharge (Day 12)	Reference Range
WBC (/mm³)	11,680 ^69^	14,500 ^70^	8,200	4,000–11,000
Platelets (/mm³)	23,000 ^71^	18,000 ^72^	145,000	150,000–450,000
Creatinine (mg/dL)	6.4 ^73^	8.78 ^74^	3.89 ^75^	0.7–1.3
Total Bilirubin (mg/dL)	26.3 ^76^	31.1 ^77^	7.5 ^7878^	0.1–1.2
Direct Bilirubin (mg/dL)	11.4 ^79^	26.0 ^80^	6.6 ^8181^	0.0–0.3
CRP (mg/L)	19.8 ^82^	27.0 ^83^	5.2	< 5.0
INR	1.2 ^84^	1.52 ^85^	1.1	0.8–1.2

## Discussion

The spirochete bacterium *Leptospira* is the cause of leptospirosis, a disease that is spread from animals to people. The infection can manifest as a mild, flu-like disease that goes away on its own or it can progress into Weil’s syndrome, a more serious condition that involves multiple organ failure and is associated with high morbidity and mortality, such as in our case report. After coming into direct touch with sick animals, the spirochetes usually enter the human body through broken skin or mucous membranes or enter indirectly through polluted settings, like soil or water tainted with infected urine [4]. There are two different clinical manifestations of leptospirosis: icteric and anicteric. In the anicteric type, symptoms include headache, cough, non-itchy rash, fever, chills, muscle soreness, lack of appetite, and diarrhea.

While the patients may continue to sometimes experience recurrent headaches, these people are likely to fully recover [5]. The more severe form of leptospirosis, known as Weil’s illness, is characterized by symptoms like jaundice, hemorrhage, and abrupt renal failure. Fever, acute kidney damage (AKI), jaundice, hemorrhage, and respiratory difficulty are some of the symptoms of this serious sickness. The illness may also affect the heart, muscles, and central nervous system (CNS) during the icteric phase. It frequently manifests as a severe, protracted illness that, if the patient survives, could linger for weeks or even months. It affects roughly 5% to 10% of infections and has a 5% to 15% fatality rate [5, 6]. 

### Immunopathogenesis of multi-organ failure

The pathogenesis of multi-organ dysfunction in leptospirosis is primarily due to widespread endothelial damage, disseminated intravascular coagulation (DIC), and severe systemic inflammation known as a “cytokine storm.” The body uses fever as a helpful but general reaction to infections and other pyrogens. It causes cytokines to be released from immune cells, which in turn stimulate brain proteins to increase body temperature. Proinflammatory and anti-inflammatory are the two primary categories into which cytokines are typically divided. IL-1, TNF-α, and IL-6 are important proinflammatory cytokines that contribute to inflammation. The pathogenesis of multi-organ dysfunction in leptospirosis is primarily due to endothelial damage, disseminated intravascular coagulation (DIC), and severe systemic inflammation [7, 8]. Multiple organs may be impacted by leptospirosis, which manifests in differing degrees of severity.

### Clinical features and differential diagnosis

The progression of the initial flu-like illness to a spectrum of symptoms including fever, extreme jaundice, and acute kidney injury was highly suggestive of severe leptospirosis (Weil’s Triad). This case is notable for the severity of hyperbilirubinemia (31.1 mg/dL), which is significantly higher than typically reported in viral hepatitis underscoring the severity of the hepatic involvement. This high severity and subsequent successful recovery despite resource limitations (lack of PCR) highlight the importance of early empirical treatment in this Middle Eastern context.

Diagnosis is further complicated by the fact that clinical symptoms might overlap with those of other diseases, such as viral hepatitis (A, B, C, E), dengue, influenza, and malaria. Unlike Dengue, which causes thrombocytopenia but typically spares the liver as severely, and Malaria, which was ruled out by blood smear, the constellation of AKI, hemorrhage, and extreme direct hyperbilirubinemia strongly points to leptospirosis.

Systemic Involvement: Up to 25% of cases result in neurological problems, most commonly aseptic meningitis. But there have also been reports of other illnesses like myelitis, cerebellitis, and cerebral bleeding [9]. There is evidence also of pulmonary involvement in leptospirosis. Since severe pulmonary hemorrhagic syndrome (SPHS), also known as severe pulmonary forms of leptospirosis (SPFL), can manifest without concomitant kidney or liver damage, it is considered a different clinical entity from Weil’s illness. Acute respiratory distress syndrome (ARDS) may develop from severe symptoms such respiratory distress or pulmonary bleeding, which are common in SPHS.- [10]. Severe leptospirosis can also lead to a life-threatening cardiac complication which can evolve to cardiogenic shock. As a result, treating leptospirosis cardiac complications particularly total atrioventricular block (TAVB) remains a significant therapeutic challenge that requires timely and effective therapy. Furthermore, a bad prognosis is indicated by the occurrence of arrhythmias, and cardiac involvement in leptospirosis is associated with a high mortality rate of 42% [4].

### Diagnostic challenges and strategy

A scoring system based on the modified Faine’s criteria has shown promise in detecting leptospirosis in low-resource environments. The 2012 updated version of the modified Faine’s criteria, which includes clinical data (Part A), epidemiological considerations (Part B), and laboratory results (Part C), was used to diagnose leptospirosis. These three factors were used to determine each patient’s Faine’s score, as shown in Table 2. Presumptive leptospirosis was defined as a score of 25 or higher from Parts A, B, and C [11].

In the case presented, the initial flu like illness of the patient progress to a wide spectrum of symptoms including fever, jaundice, and acute kidney injury, were suggestive of leptospirosis. The progression to multi-organ dysfunction, including hepatic and renal failure, thrombocytopenia, and altered mental status, pointed to a severe form of the disease: Weil’s disease. The diagnosis was confirmed by a positive leptospirosis IgM test, supporting the clinical suspicion of this neglected disease [12].

**Table 2:** Modified faine’s criteria scoring

Component	Finding in Case	Score
Part A: Clinical Findings	Myalgia (Fever, Headache)	4
	Conjunctival Suffusion	4
	Renal Failure (Creatinine > 1.4 mg/dL)	4
	Jaundice (Total Bilirubin > 2.0 mg/dL)	4
	Total Part A Score	16
Part B: Epidemiological Factors	Contact with Water/Soil/Rodent	7
	Endemic Area	0
	Total Part B Score	7
Part C: Laboratory Findings	Leukocytosis	3
	Thrombocytopenia (< 100,000/mm³)	6
	Total Part C Score	9
Total Faine’s Score (A + B+C)		32
Result	Presumptive Leptospirosis (> 25)	Confirmed

The definitive diagnosis relied on serology (IgM ELISA) because PCR testing for *Leptospira*, the gold standard in the first week (leptospiremic phase), was unavailable in our center. This delay in definitive diagnosis, inherent to serology, highlights the necessity of using clinical algorithms like the Modified Faine’s criteria to guide timely empirical treatment. Serological confirmation may take several days, which underscores the importance of empirical treatment in severe cases such as in our case where the Faine’s score was 23 from part A and B alone, and 32 (> 25) from part A, B and C (Table 2). The high mortality rate in untreated severe leptospirosis necessitates prompt initiation of antibiotics based on clinical suspicion [1].

### Therapeutic rationale

Treatment of leptospirosis typically includes antibiotic therapy. In accordance with WHO guidelines for mild cases, doxycycline is the drug of choice (100 mg orally, twice daily for 7 days), if not contraindicated. Other options include: Azithromycin (500 mg orally, once daily for 3 days), Ampicillin (500–750 mg orally, every 6 h for 7 days), Amoxicillin (500 mg orally, every 6 h for 7 days). For patients with severe disease, penicillin is the drug of choice (1.5 million units IV, every 6 h), OR Ceftriaxone (1 g IV, every 24 h). Empirical broad-spectrum antibiotics (Piperacillin-Tazobactam) and Doxycycline were initiated promptly given the patient’s septic appearance and severity of multi-organ failure.

Supportive therapies such as N-acetylcysteine (NAC) for liver protection, renal dialysis, and blood transfusions for coagulopathy are essential components of management. NAC was used as an adjunctive agent for non-acetaminophen acute liver failure, as it is theorized to improve microcirculatory blood flow and mitigate oxidative stress, though its specific efficacy in leptospirosis is an area requiring further research. Early initiation of hemodialysis can help manage severe renal dysfunction [13, 14]. 

In this case, the patient’s condition improved following the initiation of doxycycline and supportive care, including hemodialysis and nutritional support. His renal and liver functions gradually improved, and he was discharged in stable condition after 12 days of hospitalization. The prognosis of leptospirosis with multi-organ dysfunction depends on the severity of organ involvement and the timeliness of medical intervention. Mortality is high in patients who develop septic shock, renal failure, or severe hepatic dysfunction. However, with early diagnosis and aggressive supportive therapy, including antibiotics and organ-specific management, many patients can recover completely [3]. 

### Public health and prevention

A bibliographic analysis done by Wang et al. showed that over time, the study of leptospirosis has gradually progressed over years and the focus has expanded from identifying pathogens and developing diagnostics to understanding disease mechanisms and environmental influences, culminating in a multidisciplinary approach to prevention and control [15]. The Control of this fatal disease should be made as measures and policies at the source of infection (reservoir host/carrier/shedder), at the transmission route and at the level of the human host. The latter can be made by different ways such as promoting awareness and enhancing knowledge about this critical illness, give antibiotic prophylaxis by doxycycline, immunization that can be considered when there is a major public health issue, and teaching strategies to both physicians and communities [16]. Furthermore, evidence-based strategies should include aggressive reservoir control (rodent management) to limit environmental contamination.

This case, representing a successful recovery from Weil’s disease in a setting with diagnostic limitations, contributes significantly to the scarce published data from Lebanon, emphasizing the need for enhanced surveillance. In this case, the patient’s clinical recovery was slow but steady, highlighting the importance of early diagnosis and intensive supportive care in improving outcomes in severe leptospirosis and highlight the importance of raising awareness about this crucial disease to avoid the society an outbreak of such illness.

## Limitations

The primary limitations of this case report include the reliance on serology for definitive diagnosis, as molecular confirmation (PCR) was not performed due to facility constraints. This limits our ability to identify the specific infecting serovar, which could have provided further epidemiological insight. As a single case report, the generalizability of our therapeutic outcomes is limited.

## Conclusion

Leptospirosis should be considered in the differential diagnosis of patients presenting with jaundice, renal failure, thrombocytopenia, and altered mental status, particularly in endemic regions or in those with a history of potential exposure to contaminated water or soil. Early recognition and appropriate treatment, including antibiotic therapy, hemodialysis, and supportive care, are crucial in managing severe cases. Despite the potential for multi-organ dysfunction, prognosis can be favorable with prompt and aggressive treatment. We hope that preventive efforts in the world and in our country will contribute to reducing the incidence of this disease, especially given that many individuals succumb to similar symptoms without ever receiving an accurate diagnosis.

## Data Availability

The data that support the findings of this study are available on request from the corresponding author. The data are not publicly available due to privacy or ethical restrictions.
